# Association Between Medicaid Dental Payment Policies and Children’s Dental Visits, Oral Health, and School Absences

**DOI:** 10.1001/jamahealthforum.2022.3041

**Published:** 2022-09-09

**Authors:** Brandy J. Lipton, Sandra L. Decker, Brittney Stitt, Tracy L. Finlayson, Richard J. Manski

**Affiliations:** 1 Division of Health Management and Policy, School of Public Health, San Diego State University, California; 2 Center for Health Economics and Policy Studies, San Diego State University, California; 3 Division of Research and Modeling, Center for Financing, Access, and Cost Trends, Agency for Healthcare Research and Quality, Rockville, Maryland; 4 School of Dentistry, University of Maryland, Baltimore, Maryland

## Abstract

**Importance:**

Although all state Medicaid programs cover children’s dental services, less than half of publicly insured children receive recommended care.

**Objective:**

To evaluate the association between the ratio of Medicaid payment rates to dentist charges for an index of services (fee ratio) and children’s preventive dental visits, oral health, and school absences.

**Design, Setting, and Participants:**

In this cross-sectional study, a difference-in-differences analysis was conducted between September 2021 and April 2022 of 15 738 Medicaid-enrolled children and a control group of 16 867 privately insured children aged 6 to 17 years who participated in the 2016-2019 National Survey of Children’s Health. Exploratory subgroup analyses by sex and race and ethnicity were also performed. A 2-sided *P* < .05 was considered significant.

**Main Outcomes and Measures:**

Past-year preventive dental visits (at least 1 and at least 2), parent-reported excellent oral health, and number of days absent from school (at least 4 days and at least 7 days).

**Results:**

The Medicaid-enrolled sample included a weighted estimate of 51.20% boys and 48.80% girls (mean age, 11.24 years; Black, 21.65%; Hispanic, 37.75%; White, 31.45%). By weighted baseline estimates, 87% and 48% of Medicaid-enrolled children had at least 1 and at least 2 past-year dental visits, respectively, and 29% had parent-reported excellent oral health. Increasing the fee ratio by 1 percentage point was associated with percentage point increases of 0.18 in at least 1 dental visit (95% CI, 0.07-0.30), 0.27 in at least 2 visits (95% CI, 0.04-0.51), and 0.19 in excellent oral health (95% CI, 0.01-0.36). Increases in at least 2 visits were larger for Hispanic children than for White children. By weighted baseline estimates, 28% and 15% of Medicaid-enrolled children had at least 4 and at least 7 past-year school absences, respectively. Regression estimates for school absences were not statistically significant for the full sample but were estimated to be significantly reduced among girls.

**Conclusions and Relevance:**

This cross-sectional study found that more generous Medicaid payment policies were associated with significant but modest increases in children’s preventive dental visits and excellent oral health. Further research is needed to understand the potential association between policies that improve access to dental care and children’s academic success.

## Key Points

**Question:**

Are increases in the ratio of Medicaid payment rates to dentist charges for an index of services associated with improvements in children’s outcomes?

**Findings:**

This cross-sectional study used a difference-in-differences analysis to evaluate 15 738 Medicaid-enrolled and 16 867 privately insured children aged 6 to 17 years who participated in the 2016-2019 National Survey of Children’s Health. Increasing the Medicaid fee ratio was associated with significant but modest improvements in children’s dental visits and oral health and had no significant association with school absences.

**Meaning:**

More generous Medicaid dental payment policies are associated with improvements in children’s outcomes.

## Teaser

This cross-sectional study used National Survey of Children’s Health data to evaluate the association of increased Medicaid dental payment rates with children’s dental visits, oral health, and school absences.

## Introduction

Tooth decay remains one of the most common chronic diseases of childhood.^1^ Rates of untreated tooth decay are twice as high for children from low- or limited-income families compared with children from middle- or high-income families and are higher for Hispanic and non-Hispanic Black children than for non-Hispanic White children.^1^ These disparities may have implications for health and human capital development because poor oral health in childhood is associated with more school absences and lower academic achievement.^2,3,4,5,6^

State-level policies provide important leverage for reducing oral health disparities by socioeconomic status and race and ethnicity.^7^ All state Medicaid programs are required to cover a comprehensive set of preventive and restorative dental services for children, but just less than half of publicly insured children received any dental services in 2019 according to state records.^8^ Despite having coverage, some parents may struggle to find dental appointments for their children because not all dentists accept Medicaid.^9,10^ Medicaid payment rates to dentists vary at the state level but are far below private payment rates for the same services in most states.^11^ Research has suggested that increased Medicaid dental payment rates are associated with an increase in children’s dental visits.^12,13^

This study evaluated whether recent changes in the ratio of Medicaid payment rates to dentist charges for an index of services were associated with changes in children’s dental visits, oral health, and school absences. Relative to previous studies of children’s dental visits,^12,13,14^ we examined a more recent period after the major US health care reform that included the Affordable Care Act’s Medicaid expansion and the inclusion of dental as an essential health benefit for children. In addition, to our knowledge, the present study is among the first to assess whether Medicaid dental payment rates are associated with improvements in children’s oral health and school absences and to document whether associations differ by sex and race and ethnicity.

## Methods

### Data, Outcomes, and Primary Exposure

In this cross-sectional study conducted between September 2021 and April 2022, we used data from the 2016-2019 National Survey of Children’s Health (NSCH), a nationally representative, annually repeated cross-sectional mail and web-based survey.^15^ Response rates for the 2016-2019 surveys ranged from a minimum of 37.4% (2017) to a maximum of 43.1% (2018).^16^ The main binary outcome variables included having at least 1 past-year preventive dental visit, at least 2 past-year visits, parent-reported excellent oral health status, at least 4 school absences, and at least 7 school absences. The question about dental visits was as follows: “Did this child see a dentist or other oral health care provider for preventive dental care, such as check-ups and dental cleanings, dental sealants, or fluoride treatments?” In exploratory analyses, we also assessed binary indicators for specific past-year preventive services, including dental cleaning, toothbrushing instructions, x-rays, fluoride treatment, and dental sealants. Only children who had a past-year preventive dental visit responded to questions about the specific preventive services they received.

The primary exposure variable was the continuous ratio of Medicaid fees to dentist charges for an index of services, which we refer to hereafter as the fee ratio. Medicaid rates were obtained from fee schedules posted on state websites, but this information was unavailable for Colorado, Delaware, Idaho, and Tennessee; thus, these states were omitted from our analysis. Dentist charges for the same services were used as a denominator to deflate Medicaid payment rates and were obtained from the American Dental Association. eAppendix 1 in the Supplement describes the fee ratio in more detail.

### Statistical Analysis

We estimated the association between the fee ratio and school-aged children’s outcomes using a difference-in-differences approach that leveraged state-level changes in the continuous fee ratio during the 2016-2019 study period. Unlike the use of a binary policy variable, this modeling choice considered all changes in the fee ratio, regardless of their magnitude, and allowed greater changes in the fee ratio to have larger associations with the outcomes. Similar to related research,^12,13,14^ this approach compared Medicaid-enrolled and privately insured children in states with larger and smaller changes in the fee ratio before and after these changes occurred at any time during our study period. Privately insured children with family incomes up to 300% of the federal poverty level were included as a control group because they were not expected to be directly affected by Medicaid policies. The final sample included 15 738 Medicaid-enrolled children and 16 867 privately insured children who were aged 6 to 17 years at the time of the survey.

Corresponding weighted sample sizes were approximately 56 million Medicaid-enrolled children and 37 million privately insured children. eAppendix 2 in the Supplement includes additional information on the sample and exclusion criteria.

The independent variable of interest was the interaction between the fee ratio and an indicator for Medicaid coverage. Models controlled for state indicators to account for time-invariant state-level characteristics that could be associated with Medicaid payment policies and outcomes and year indicators to account for national outcome trends during the study period.

We controlled for parent-reported child and household characteristics, including child age, sex, race and ethnicity, whether US born, number of children in the household, highest adult educational attainment, and primary caregiver marital and employment status. We recoded race and ethnicity into 4 mutually exclusive categories, including Hispanic, non-Hispanic Black, non-Hispanic White (reference), and non-Hispanic other race. The other race category included American Indian or Alaska Native, Asian, Native Hawaiian and Other Pacific Islander, and 2 or more races. We also included the state-by-year unemployment rate, Medicaid managed care penetration rate, dentist supply per capita, and Medicaid income eligibility limit for working parents and interacted each of these variables with the Medicaid indicator. eAppendix 3 in the Supplement describes the model in more detail. We used sample-weighted linear probability models to avoid attenuation bias in nonlinear models with a substantial number of fixed effects, and errors were clustered at the state level.^17,18^

We conducted exploratory analyses by child sex and race and ethnicity by estimating models with a complete set of interactions between the subgroup characteristics and regression controls. The 3-way interaction among each subgroup indicator, the fee ratio, and the Medicaid coverage indicator was used to determine whether associations differed for that subgroup relative to the reference group. The relative magnitude of associations by sex and race and ethnicity was a priori ambiguous given the dearth of research examining subgroup differences in the association between Medicaid dental payment policies and children’s outcomes and the myriad factors that could strengthen or weaken these associations. For example, associations could differ by subgroup because of differences in access to dentists, unmet dental care needs, parental health literacy, awareness of a child’s health needs, or propensities to invest in a child’s health.

This research followed the Strengthening the Reporting of Observational Studies in Epidemiology (STROBE) reporting guidelines for cross-sectional studies by defining all outcomes and exposures; describing the sample inclusion criteria; describing statistical methods, including those used to evaluate subgroups and interactions; and reporting sensitivity analyses. The San Diego State University institutional review board determined that this study was exempt from providing informed consent because it was not human participants research.

We conducted several tests of the robustness of our main results. First, we evaluated whether outcomes were trending similarly for Medicaid-enrolled and privately insured children before the fee ratio changes in 2 ways. We visually inspected outcome trends for Medicaid-enrolled and privately insured children in 9 states that first experienced a year-over-year change in the fee ratio of at least 2 percentage points in 2018 or 2019. We then conducted a more rigorous regression-based test by including the future change in the fee ratio and its interaction with the Medicaid indicator as explanatory variables in our regression models. A significant coefficient on this interaction term would suggest that outcome changes may have preceded fee ratio changes, indicating a violation of parallel trends. Second, recent evidence has suggested that difference-in-differences estimates may be biased when states change their policies at different times.^19^ We assessed this possibility by estimating our main model separately for states with a change in the fee ratio in each year from 2017 to 2019, again defined as a year-over-year change of at least 2 percentage points. Third, we restricted the sample to children residing in 28 states that provided children’s dental services on a mainly fee-for-service basis.^11^ Fourth, we estimated models that replaced the continuous fee ratio with binary indicators for having a ratio above vs below the 75th and 50th percentiles of the distribution, respectively. Finally, we tested the sensitivity of the results to using the inflation-adjusted Medicaid fee index, including a control for family income, excluding 6 states with a change in Medicaid adult dental coverage policy, restricting the sample to US-born children, estimating logit models, and excluding the privately insured control group. These sensitivity tests are discussed in the Results, and further details are given in eAppendix 4 in the Supplement. A 2-sided *P* < .05 was considered significant. All statistical analyses were conducted with Stata/MP, version 15.0 (StataCorp) software.

## Results

### Sample Descriptive Statistics and Outcome Trends

Table 1 lists the sample descriptive statistics using weighted estimates of rates of each characteristic for Medicaid-enrolled and privately insured children. Among the Medicaid-enrolled sample, 51.20% were boys and 48.80% were girls (mean age, 11.24 years; Black, 21.65%; Hispanic, 37.75%; White, 31.45%; other races [American Indian or Alaska Native, Asian, Native Hawaiian or Other Pacific Islander, and ≥2 races], 9.15%). The majority of children were US born (95.16%), and more than half (55.43%) resided in a home where the highest adult educational attainment was a high school diploma or General Educational Development certification or less education. There were significant differences between the Medicaid-enrolled and privately insured samples by race and ethnicity, adult educational attainment, number of children living in the household, and primary caregiver marital and employment status (all *P* < .001; all supporting data reported in Table 1). However, we did not find any evidence that the fee ratio was associated with the sample’s composition (eTable 1 in the Supplement).

**Table 1.** Sample Descriptive Statistics^a^

Characteristic	Weighted %		*P* value for difference
	Medicaid insured	Privately insured	
Mean age, years	11.24	11.65	<.001
Sex			
Male	51.20	50.41	.37
Female	48.80	49.59	
Race and ethnicity			<.001
Hispanic	37.75	24.41	
Non-Hispanic			
Black	21.65	13.23	
White	31.45	52.67	
Other^b^	9.15	9.69	
US born	95.16	95.43	.64
No. of children in household			<.001
1	24.10	24.09	
2	32.97	38.68	
≥3	42.92	37.23	
Highest adult educational attainment			<.001
High school diploma, GED, or less	55.43	25.24	
Some college or associate’s degree	28.36	28.59	
College or more education	16.21	46.17	
Primary caregiver married or living with partner	64.72	80.46	<.001
Primary caregiver employed	77.47	94.58	<.001

Abbreviation: GED, General Educational Development certification.

^a^ Estimates shown are weighted rates for the Medicaid- and privately insured samples of families interviewed during the 2016-2019 National Survey of Children’s Health.

^b^ The other race category includes American Indian or Alaska Native, Asian, Native Hawaiian or Other Pacific Islander, and 2 or more races.

**Figure 1.**
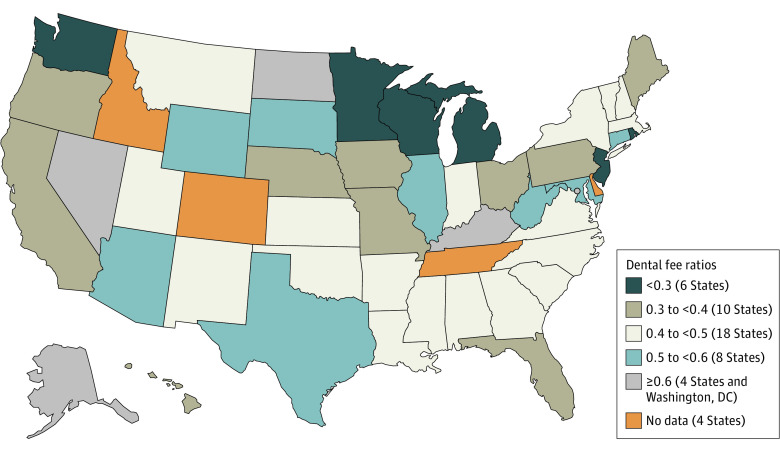
Medicaid Fee-for-Service Reimbursement as a Percentage of Fees Charged by Dentists by State in 2019 Analysis of state fee schedules from state Medicaid websites and the 2018 and 2020 American Dental Association Survey of Dental Fees.^20,21^ More details on the construction of the fee ratio are available in eAppendix 1 in the Supplement.

Figure 1 summarizes the 2019 state-level fee ratios.^20,21^ Although most state ratios were between 0.3 and 0.6, 6 states had a ratio less than 0.3, and 4 states and the District of Columbia had a ratio of 0.6 or greater. Most states experienced decreases between 2016 and 2019, although many year-over-year changes were no more than 1 to 3 percentage points in absolute value (eTable 2 in the Supplement). Twenty states had a year-over-year change of at least 2 percentage points. We used observations for Medicaid-enrolled children in these 20 states during the survey years preceding fee ratio changes to calculate baseline outcome rates (Table 2 and Table 3).

**Table 2.** Difference-in-Differences Estimates of the Association Between the Medicaid Fee Ratio and Children’s Preventive Dental Visits: 2016-2019 National Survey of Children’s Health^a^

Outcome	No. of Medicaid-insured children	Weighted baseline %	Difference-in-differences estimate (95% CI)	*P* value
**Any preventive dental visit**				
All	15 650	87	0.18 (0.07 to 0.30)	.002
Subgroup analysis by race and ethnicity				
Hispanic	3095	87	0.17 (0.00 to 0.34)	.047
Non-Hispanic				
Black	2129	88	0.07 (−0.29 to 0.43)	.70
White [Reference]	8268	87	0.19 (0.04 to 0.34)	.014
Other^b^	2158	84	−0.14 (−0.42 to 0.15)	.34
Subgroup analysis by sex				
Male [Reference]	8139	87	0.22 (0.05 to 0.39)	.02
Female	7511	87	0.14 (−0.05 to 0.32)	.14
**At least 2 preventive dental visits**				
All	15 650	48	0.27 (0.04 to 0.51)	.02
Subgroup analysis by race and ethnicity				
Hispanic	3095	49	0.59 (0.19 to 0.99)^c^	.005
Non-Hispanic				
Black	2129	50	0.17 (−0.28 to 0.61)	.46
White [Reference]	8268	47	0.14 (−0.16 to 0.43)	.37
Other^b^	2158	43	−0.00 (−0.38 to 0.37)	>.99
Subgroup analysis by sex				
Male [Reference]	8139	48	0.22 (−0.09 to 0.53)	.16
Female	7511	49	0.35 (0.02 to 0.68)	.04

^a^ Estimates are in terms of percentage points. Weighted baseline rates are for Medicaid-enrolled children in 20 states with a year-over-year fee ratio change of at least 2 percentage points in absolute value and calculated before this change occurred, as described in the text. Difference-in-differences estimates represent the effect of a 1 percentage point increase in the fee ratio. Models include state and year as fixed effects in addition to child and time-varying state-level controls (unemployment rate, Medicaid managed care penetration rate, dentist supply per capita, and Medicaid income eligibility limit for working parents). Estimates are weighted and model errors clustered at the state level. Subgroup estimates and tests for differences between each subgroup and the reference group are from models with a complete set of interactions between the subgroup indicators and the main analysis controls.

^b^ The other race category includes American Indian or Alaska Native, Asian, Native Hawaiian or Other Pacific Islander, and 2 or more races.

^c^ Significant difference between the subgroup and reference group (*P* < .05).

**Table 3.** Difference-in-Differences Estimates of the Association Between the Medicaid Fee Ratio and Children’s Oral Health and School Absences: 2016-2019 National Survey of Children’s Health^a^

Outcome	No. of Medicaid-insured children	Weighted baseline %	Difference-in-differences estimate (95% CI)	*P* value
**Excellent condition of teeth**				
All	15 686	29	0.19 (0.01 to 0.36)	.04
Subgroup analysis by race and ethnicity				
Hispanic	3105	26	0.31 (0.11 to 0.50)	.003
Non-Hispanic				
Black	2140	33	0.25 (−0.41 to 0.92)	.45
White [Reference]	8283	31	0.05 (−0.19 to 0.28)	.70
Other^b^	2158	27	−0.13 (−0.44 to 0.18)	.41
Subgroup analysis by sex				
Male [Reference]	8159	27	0.09 (−0.10 to 0.29)	.35
Female	7527	31	0.26 (0.03 to 0.49)	.03
**At least 4 school absences**				
All	15 458	28	−0.07 (−0.21 to 0.06)	.29
Subgroup analysis by race and ethnicity				
Hispanic	3062	25	−0.03 (−0.35 to 0.29)	.83
Non-Hispanic				
Black	2095	24	0.14 (−0.21 to 0.49)	.42
White [Reference]	8171	32	−0.08 (−0.28 to 0.12)	.42
Other race	2130	36	−0.20 (−0.40 to 0.01)	.06
Subgroup analysis by sex				
Male [Reference]	8040	26	−0.01 (−0.22 to 0.19)	.89
Female	7418	30	−0.14 (−0.30 to 0.03)	.10
**At least 7 school absences**				
All	15 458	15	−0.09 (−0.19 to 0.00)	.06
Subgroup analysis by race and ethnicity				
Hispanic	3062	13	−0.24 (−0.55 to 0.08)	.13
Non-Hispanic				
Black	2095	12	0.01 (−0.22 to 0.23)	.96
White [Reference]	8171	18	−0.04 (−0.17 to 0.10)	.60
Other^b^	2130	16	−0.09 (−0.36 to 0.19)	.54
Subgroup analysis by sex				
Male [Reference]	8040	15	0.02 (−0.09 to 0.14)	.71
Female	7418	14	−0.22 (−0.35 to −0.09)^c^	.001

^a^ Estimates shown are in terms of percentage points. Weighted baseline rates are for Medicaid-enrolled children in 20 states with a year-over-year fee ratio change of at least 2 percentage points in absolute value and calculated before this change occurred, as described in the text. Difference-in-differences estimates represent the effect of a 1 percentage point increase in the fee ratio. Models include state and year as fixed effects in addition to the child and time-varying state-level controls (unemployment rate, Medicaid managed care penetration rate, dentist supply per capita, and Medicaid income eligibility limit for working parents). Estimates are weighted and model errors clustered at the state level. Subgroup estimates and tests for differences between each subgroup and the reference group are from models with a complete set of interactions between the subgroup indicators and the main analysis controls.

^b^ The other race category includes American Indian or Alaska Native, Asian, Native Hawaiian or Other Pacific Islander, and 2 or more races.

^c^ Significant difference between the subgroup and reference group (*P* < .01).

**Figure 2.**
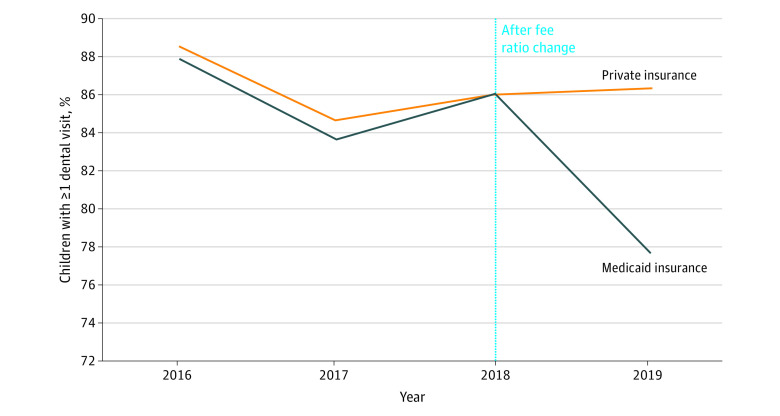
Trends in at Least 1 Dental Visit in States With a 2018-2019 Fee Ratio Change Outcome rates are weighted using sampling weights available from the 2016-2019 National Survey of Children’s Health. The sample includes children in the main study sample who resided in 1 of 9 states that first had a year-over-year fee ratio change of at least 2 percentage points in 2018 or 2019. These states were Alaska, Arizona, Connecticut, Mississippi, Montana, North Carolina, Oregon, South Dakota, and Utah. The sample includes 3420 Medicaid-enrolled children and 2923 privately insured children.

Figure 2 shows trends in having at least 1 dental visit for Medicaid-enrolled and privately insured children in the 9 states that first had a year-over-year change of at least 2 percentage points in 2018 or 2019. This analysis included states that experienced both decreases (6 states) and increases (3 states) in the fee ratio during our study period to maximize the sample size for assessment of pretrends. In this descriptive analysis, trends seemed similar for Medicaid-enrolled and privately insured children before 2018, after which dental visit rates decreased for Medicaid-enrolled children only. This finding was expected because the fee ratio decreased in 6 of these 9 states. Analogous graphs for the other outcomes are included in eFigures 1 to 4 in the Supplement. It is important to note that this analysis was based on a limited number of preperiods because of our relatively short study period and that estimates had wide 95% CIs. Nonetheless, inspection of these graphs did not suggest a rejection of the parallel trends assumption.

### Main Regression Results

Table 2 presents regression-adjusted difference-in-differences estimates for the dental visit outcomes. The weighted baseline estimates indicated that 87% and 48% of Medicaid-enrolled children had at least 1 and at least 2 past-year dental visits, respectively. A 1 percentage point increase in the fee ratio was associated with percentage point increases of 0.18 (95% CI, 0.07-0.30) and 0.27 (95% CI, 0.04-0.51), respectively, in these outcomes. Among the specific preventive services that we assessed as secondary outcome measures, we estimated significant increases in dental cleanings, toothbrushing instructions, and fluoride treatments (eTable 3 in the Supplement).

In exploratory analyses by race and ethnicity, we estimated positive associations between the fee ratio and at least 1 past-year dental visit for Hispanic (0.17; 95% CI, 0.00-0.34) and White (0.19; 95% CI, 0.04-0.34) children and nonsignificant associations for Black children (0.07; 95% CI, –0.29 to 0.43) and children of other races (–0.14; 95% CI, –0.42 to 0.15), but between-group differences were not significant. The estimate for Black children was significant when considering the larger sample of children aged 0 to 17 years (eTable 4 in the Supplement). The association between the fee ratio and at least 2 past-year dental visits was only significant for Hispanic children (0.59; 95% CI, 0.19-0.99), and this estimate was significantly different from that for White children. The fee ratio was associated with increases in dental visits among male and female children and were not significantly different from each other.

Table 3 presents difference-in-differences estimates for the parent-reported excellent oral health and school absence outcomes. The weighted baseline estimates indicated that 29% of Medicaid-enrolled children had excellent oral health and that 28% and 15% had at least 4 and at least 7 school absences, respectively. A 1 percentage point increase in the fee ratio was associated with a significant increase in excellent oral health (0.19; 95% CI, 0.01-0.36) and modest decreases in school absences (at least 4: –0.07 [95% CI, –0.21 to 0.06]; at least 7: –0.09 [95% CI, –0.19 to 0.00]) that were not significant among the full sample.

In exploratory subgroup analyses, improvements in excellent oral health were only significant for Hispanic children (0.31; 95% CI, 0.11-0.50) and girls (0.26; 95% CI, 0.03-0.49) (Table 3). However, none of the between-group differences were significant. In terms of school absences, none of the estimates by race and ethnicity were significant, but analyses by child sex suggested that the fee ratio was associated with a significant decrease in at least 7 school absences among girls (–0.22; 95% CI, −0.35 to −0.09). This latter result was significantly different from boys.

### Sensitivity Analyses

Our regression-based analysis did not suggest significant differential preexisting outcome trends for any of the outcomes considered (eTable 5 in the Supplement). We also did not find evidence that differences in the timing of state-level fee ratio changes led to biased results (eTable 6 in the Supplement). Restricting the sample to states with fee-for-service dental programs resulted in somewhat larger associations, as expected (eTable 7 in the Supplement). Results were qualitatively consistent when we replaced the fee ratio with indicators for having a ratio greater than the 75th or 50th percentiles of the distribution (eTable 8 in the Supplement) or with the inflation-adjusted Medicaid fee index (eTable 9 in the Supplement) and when we estimated logit models instead of linear probability models (eTable 10 in the Supplement). Results were also similar when we included the additional controls and considered the alternative sample exclusions (eTable 11 in the Supplement). Finally, estimates were qualitatively consistent but had larger SEs and were generally no longer statistically significant when excluding the privately insured control group (eTable 12 in the Supplement).

## Discussion

The American Academy of Pediatric Dentistry recommends that children visit the dentist every 6 months.^22^ Although approximately 87% of publicly insured children had at least 1 past-year dental visit at baseline in our analysis of NSCH data, less than half met this recommendation. Consistent with previous research,^12,13,14^ we found in this cross-sectional study that recent increases in the ratio of Medicaid payment rates to dentist charges for an index of services were positively associated with children’s dental visit rates. Most comparable to our analysis, one study found that a 10 percentage point increase in the ratio of Medicaid to private dental fees was associated with a 1.5 percentage point increase in at least 1 past-year dental visit,^13^ which is similar to our estimate of a 1.8 percentage point increase (0.18 × 10) or a 2.0% change relative to the baseline rate. We also found evidence of a significant increase in parent-reported excellent oral health, although, in line with our analysis of dental visits, improvements were modest.

In exploratory analyses by race and ethnicity, we found that increasing the Medicaid fee ratio had the largest associations with dental visits and oral health among Hispanic children, although only the estimate for at least 2 past-year dental visits was significantly different from White children. Larger associations among Hispanic children could be due to differences in neighborhood characteristics, such as local dentist supply and access to federally qualified health centers. One study found that dental care use was more responsive to metropolitan area residence among Medicaid-enrolled Hispanic children than White children.^23^ Larger sample sizes to further investigate subgroup differences and research to enhance understanding of the role of specific neighborhood, familial, and local policy factors in children’s dental care access are important considerations for future studies.

Poor oral health in childhood has been associated with more school absences and lower grades,^2,3,4,5,6,24^ which raises the possibility that policies that improve children’s oral health may also lead to improvements in academic outcomes. However, it is also plausible that children may incur additional school absences to receive dental treatment. Because most existing research used a correlational study design, it is difficult to disentangle the influence of poor oral health from other associated factors, such as socioeconomic status and health literacy.^6^ We tested whether a policy with an established association with dental visits was also associated with school absences by using a cross-sectional study with a difference-in-differences research design.

Estimates for the full sample suggest a negative but modest correlation between the fee ratio and school absences that was not statistically significant. In exploratory subgroup analyses, we found that girls experienced a significant decrease in having at least 7 school absences, which suggests the possibility of a meaningful role for oral health in promoting school attendance that merits further attention. It is plausible that male and female children tend to communicate differently about health concerns, such as oral pain, which may lead to disparities in parental awareness of children’s unmet oral health needs. Furthermore, parents may have different propensities to allow male and female children to stay home from school because of health complaints or may be differentially inclined to invest in the health of male and female children. Gaining a clearer understanding of the extent to which these factors may explain male-female differences in the effectiveness of policies that target children’s dental care access is an area for further research.

### Limitations

This study had several limitations. First, changes in the fee ratio were modest during the short period of our study relative to previous research examining historical changes during the period from 2000 to 2009.^12,13^ Therefore, our power to detect significant changes in some of the outcomes and for smaller subgroups, including Black children and children of other races, was also limited.

Second, similar to other national surveys, outcomes were based on parent reports, and estimated dental visit rates were high relative to state records.^8^ Third, the NSCH included information on school absences, but we could not assess other measures of academic success, such as test scores or course grades. Similarly, the survey included information on preventive dental care, but we were not able to assess children’s receipt of restorative services. Finally, it was not possible to rule out bias due to unobserved confounding factors given the observational nature of our study, and we were not able to test whether the association between the fee ratio and children’s outcomes was moderated by dentist participation in Medicaid, as has been found in previous research.^25^

## Conclusions

In this cross-sectional study using recent national data, we found that more generous Medicaid payment policies were associated with improvements in children’s dental visit rates and oral health, with some evidence of larger benefits for Hispanic children and girls. Our overall findings are in line with past research that concluded that increasing Medicaid payment rates to dentists was associated with a relatively modest increase in children’s dental visits. Nonetheless, the finding of larger associations for Hispanic children may indicate a role for Medicaid payment policy in reducing health care disparities by ethnicity. Furthermore, the potential for improvements in children’s oral health and reductions in school absences may be important considerations in state assessments of Medicaid fee schedules.

## Notes

Supplement. **eAppendix 1.** Description of Fee Ratio Construction **eAppendix 2.** Sample Exclusions and Sample Sizes **eAppendix 3.** Description of Model Controls and Outcome Variables **eAppendix 4.** Description of Supplementary Tables and Figures **eTable 1.** Association Between the Fee Ratio and Sample Characteristics, National Survey of Children’s Health 2016-2019 **eTable 2.** State-Level Fee Ratio Changes, 2016-2019 **eTable 3.** Difference-in-Differences Estimates of the Association Between the Fee Ratio and Specific Preventive Services, National Survey of Children’s Health 2016-2019 **eTable 4.** Difference-in-Differences Estimates of the Association Between the Fee Ratio and Children’s Preventive Dental Visits for Children of All Ages, National Survey of Children’s Health 2016-2019 **eTable 5.** Test for Preexisting Differences in Outcome Trends, National Survey of Children’s Health 2016-2019 **eTable 6.** Difference-in-Differences Estimates of the Association Between the Fee Ratio and Preventive Dental Visits, Oral Health, and School Absences by Year of Policy Change, National Survey of Children’s Health 2016-2019 **eTable 7.** Difference-in-Differences Estimates of the Association Between the Fee Ratio and Preventive Dental Visits, Oral Health, and School Absences in States With a Fee-for-Service Dental Program, National Survey of Children’s Health 2016-2019 **eTable 8.** Difference-in-Differences Estimates of the Association Between the Fee Percentile and Preventive Dental Visits, Oral Health, and School Absences, National Survey of Children’s Health 2016-2019 **eTable 9.** Difference-in-Differences Estimates of the Association Between the Inflation-Adjusted Medicaid Fee Index and Preventive Dental Visits, Oral Health, and School Absences, National Survey of Children’s Health 2016-2019 **eTable 10.** Difference-in-Differences Estimates of the Association Between the Fee Ratio and Preventive Dental Visits, Oral Health, and School Absences, Logit Regression Models, National Survey of Children’s Health 2016-2019 **eTable 11.** Difference-in-Differences Estimates of the Association Between the Fee Ratio and Preventive Dental Visits, Oral Health, and School Absences, Various Controls and Sample Exclusions, National Survey of Children’s Health 2016-2019 **eTable 12.** Difference-in-Differences Estimates of the Association Between the Fee Ratio and Preventive Dental Visits, Oral Health, and School Absences Excluding the Privately Insured Control Group, National Survey of Children’s Health 2016-2019 **eFigure 1.** Trends in at Least 2 Dental Visits in States With a 2018-2019 fee Ratio Change **eFigure 2.** Trends in Excellent Oral Health in States With a 2018-2019 Fee Ratio Change **eFigure 3.** Trends in at Least 4 School Absences in States With a 2018-2019 Fee Ratio Change **eFigure 4.** Trends in at least 7 School Absences in States With a 2018-2019 Fee Ratio Change
