# Supplementary material for: Association Between Medicaid Dental Payment Policies and Children’s Dental Visits, Oral Health, and School Absences
Source: JAMA Health Forum. 2022 Sep 9;3(9):e223041. doi: 10.1001/jamahealthforum.2022.3041 (PMC9463610; doi:10.1001/jamahealthforum.2022.3041)
Supplement: Supplement. — eAppendix 1. Description of Fee Ratio Construction eAppendix 2. Sample Exclusions and Sample Sizes eAppendix 3. Description of Model Controls and Outcome Variables eAppendix 4. Description of Supplementary Tables and Figures eTable 1. Association Between the Fee Ratio and Sample Characteristics, National Survey of Children’s Health 2016-2019 eTable 2. State-Level Fee Ratio Changes, 2016-2019 eTable 3. Difference-in-Differences Estimates of the Association Between the Fee Ratio and Specific Preventive Services, National Survey of Children’s Health 2016-2019 eTable 4. Difference-in-Differences Estimates of the Association Between the Fee Ratio and Children’s Preventive Dental Visits for Children of All Ages, National Survey of Children’s Health 2016-2019 eTable 5. Test for Preexisting Differences in Outcome Trends, National Survey of Children’s Health 2016-2019 eTable 6. Difference-in-Differences Estimates of the Association Between the Fee Ratio and Preventive Dental Visits, Oral Health, and School Absences by Year of Policy Change, National Survey of Children’s Health 2016-2019 eTable 7. Difference-in-Differences Estimates of the Association Between the Fee Ratio and Preventive Dental Visits, Oral Health, and School Absences in States With a Fee-for-Service Dental Program, National Survey of Children’s Health 2016-2019 eTable 8. Difference-in-Differences Estimates of the Association Between the Fee Percentile and Preventive Dental Visits, Oral Health, and School Absences, National Survey of Children’s Health 2016-2019 eTable 9. Difference-in-Differences Estimates of the Association Between the Inflation-Adjusted Medicaid Fee Index and Preventive Dental Visits, Oral Health, and School Absences, National Survey of Children’s Health 2016-2019 eTable 10. Difference-in-Differences Estimates of the Association Between the Fee Ratio and Preventive Dental Visits, Oral Health, and School Absences, Logit Regression Models, National Survey of Children’s Health 2016-2019 eTable [file jamahealthforum-e223041-s001.pdf]

Supplemental Online Content

Lipton BJ, Decker SL, Stitt B, Finlayson TL, Manski RJ. Association between Medicaid dental payment policies and children's dental visits, oral health, and school absences. *JAMA Health Forum*. 2022;3(9):e223041. doi:10.1001/jamahealthforum.2022.3041

eAppendix 1. Description of Fee Ratio Construction

eAppendix 2. Sample Exclusions and Sample Sizes

eAppendix 3. Description of Model Controls and Outcome Variables

eAppendix 4. Description of Supplementary Tables and Figures

eTable 1. Association Between the Fee Ratio and Sample Characteristics, National Survey of Children's Health 2016-2019

eTable 2. State-Level Fee Ratio Changes, 2016-2019

eTable 3. Difference-in-Differences Estimates of the Association Between the Fee Ratio and Specific Preventive Services, National Survey of Children's Health 2016-2019

eTable 4. Difference-in-Differences Estimates of the Association Between the Fee Ratio and Children's Preventive Dental Visits for Children of All Ages, National Survey of Children's Health 2016-2019

eTable 5. Test for Preexisting Differences in Outcome Trends, National Survey of Children's Health 2016-2019

eTable 6. Difference-in-Differences Estimates of the Association Between the Fee Ratio and Preventive Dental Visits, Oral Health, and School Absences by Year of Policy Change, National Survey of Children's Health 2016-2019

eTable 7. Difference-in-Differences Estimates of the Association Between the Fee Ratio and Preventive Dental Visits, Oral Health, and School Absences in States With a Fee-for-Service Dental Program, National Survey of Children's Health 2016-2019

eTable 8. Difference-in-Differences Estimates of the Association Between the Fee Percentile and Preventive Dental Visits, Oral Health, and School Absences, National Survey of Children's Health 2016-2019

eTable 9. Difference-in-Differences Estimates of the Association Between the Inflation-Adjusted Medicaid Fee Index and Preventive Dental Visits, Oral Health, and School Absences, National Survey of Children's Health 2016-2019

eTable 10. Difference-in-Differences Estimates of the Association Between the Fee Ratio and Preventive Dental Visits, Oral Health, and School Absences, Logit Regression Models, National Survey of Children's Health 2016-2019

eTable 11. Difference-in-Differences Estimates of the Association Between the Fee Ratio and Preventive Dental Visits, Oral Health, and School Absences, Various Controls and Sample Exclusions, National Survey of Children's Health 2016-2019

© 2022 Lipton BJ et al. *JAMA Health Forum.*

eTable 12. Difference-in-Differences Estimates of the Association Between the Fee Ratio and Preventive Dental Visits, Oral Health, and School Absences Excluding the Privately Insured Control Group, National Survey of Children's Health 2016-2019

eFigure 1. Trends in at Least 2 Dental Visits in States With a 2018-2019 fee Ratio Change

eFigure 2. Trends in Excellent Oral Health in States With a 2018-2019 Fee Ratio Change

eFigure 3. Trends in at Least 4 School Absences in States With a 2018-2019 Fee Ratio Change

eFigure 4. Trends in at least 7 School Absences in States With a 2018-2019 Fee Ratio Change

This supplemental material has been provided by the authors to give readers additional information about their work.

eAppendix 1. Description of Fee Ratio Construction

We constructed an index of Medicaid fees using the most commonly billed codes for children's dental visits according to a recent American Dental Association (ADA) analysis. Services included in order of their weight in the index were periodic oral evaluation – established patient (D0120), child prophylaxis (D1120), adult prophylaxis (D1110), topical fluoride application (D1208), sealant application (D1351), bitewing radiographic images (D0272 and D0274), topical application of fluoride varnish (D1206), and periapical intraoral radiographic images (D0220 and D0230). Medicaid payment rates for each state and year were obtained from fee schedules posted on state Medicaid websites. Since we only observed calendar year in the NSCH, we weighted state-level payment rates for each service by the number of months they were in effect in each year.

To account for geographic differences in the market for dental services and changes over time, we deflated this index by dentist-reported charges for the same services from the 2016, 2018, and 2020 ADA Survey of Dental Fees. The ADA's survey reports fees for 9 geographic regions in each year. We used linear interpolation between years to compute these charges by region for 2017 and 2019.

eAppendix 2. Sample Exclusions and Sample Sizes

The sample included publicly insured children and a control group of privately insured children. Public health insurance coverage included "Medicaid, medical assistance, or any kind of governmental assistance plan for those with low incomes or a disability." Children who reported public health insurance alone or in combination with a second source of coverage were considered publicly insured. The sample was restricted to children ages 6-17 since our analysis examined school absences. We also limited the sample to children with family incomes up to

© 2022 Lipton BJ et al. *JAMA Health Forum.*

300% of the federal poverty level to increase comparability between the treatment group of Medicaid-enrolled children and the control group including privately insured children. Before making any additional exclusions, the sample included 36,921 children, including 17,866 publicly insured children and 19,055 privately insured children.

We excluded children who resided in Colorado, Delaware, Idaho, and Tennessee because Medicaid payment rate information was not available for these states (3,041 children). After making this exclusion, the sample included 33,880 children, including 16,380 children with public health insurance and 17,500 children with private health insurance. We also excluded 1,275 children (3.8%) who had missing information for any of the demographic characteristics included as controls in our regression models. The final analysis sample included 32,605 children, including 15,738 with public health insurance and 16,867 with private health insurance.

eAppendix 3. Description of Regression Model and Outcome Variables

Our main regression model was of the following form:

$$\boldsymbol{Y_{ist} = \beta_1 feeratio_{st} + \beta_2 Medicaid_{it} + \beta_3 feeratio_{st} \times Medicaid_{it} + X_{ist} + \gamma_s + \tau_t + \varepsilon_{ist}}$$

Where Y_{ist} is the outcome of interest for child i, who resides in state s, and is observed during interview year t; $feeratio_{st}$ is the state-by-year continuous fee ratio, and $Medicaid_{it}$ is a binary indicator for Medicaid coverage. X_{ist} represents child and household characteristics and state-by- year policy variables, γ_s represents state indicators, and τ_t represents year indicators**.** The term ε_{ist} is an error term, which is clustered by state. All models are weighted using sampling weights available from the National Survey of Children's Health, and models are estimated using linear probability models.

The coefficient of interest is $\boldsymbol{\beta_3}$, which provides the difference-in-differences estimate for the interaction term between the continuous fee ratio and the indicator for Medicaid coverage.

Child and household characteristics included as controls are child age in years, sex (male vs. female), race and ethnicity (Hispanic, Non-Hispanic Black, Non-Hispanic Other Races vs. Non-Hispanic White), an indicator for a child being born in the United State, number of minor children in the household (1, 2 vs. 3 or more), highest adult educational attainment (high school diploma/GED or less educational attainment, some college education vs. college or more education), parent or caregiver marital status (married or living with a partner vs. never married, divorced, separated, or widowed), and parent or caregiver employment status (employed vs. not employed). If there was more than one parent or caregiver in the household, then the marital and employment controls indicated whether either person held that status.

In addition, models controlled for the state-by-year unemployment rate (Bureau of Labor Statistics), Medicaid managed care penetration rate – any managed care (Centers for Medicare and Medicaid Services and the Kaiser Family Foundation), dentists per capita (Area Health Resources File), and Medicaid eligibility limits for working parents (Kaiser Family Foundation).

Outcome variables included children's past-year preventive dental visits (at least 1 and at least 2), excellent oral health, and school absences (at least 4 and at least 7). Response options for the question about children's oral health status included excellent, very good, good, fair or poor. Response options for the question about children's school absences included none, 1-3 days, 4-6 days, 7-10 days, and 11 or more days.

eAppendix 4. Description of Supplementary Tables and Figures

Table S1 presents associations between the fee ratio and each sample characteristic to test if the fee ratio is systematically correlated with the sample's composition. A separate regression was run for each sample characteristic as an outcome variable. The regressors included the fee ratio and its interaction with the Medicaid coverage indicator, state, and year fixed effects.

© 2022 Lipton BJ et al. *JAMA Health Forum.*

Regressions were weighted and model errors were clustered at the state level, as in our main analysis. Significant coefficients on the interaction term between the fee ratio and the Medicaid coverage indicator would suggest that the composition of Medicaid-enrolled children was changing relative to that for privately-insured children with changes to the fee ratio, which could bias our main difference-in-differences results. As shown in Table S1, none of the associations with the sample characteristics were statistically significant at conventional levels.

Table S2 provides the 2016 and 2019 fee ratios, and the percentage-point change from 2016 to 2019, by state. As described in the text, most fee ratio changes were declines, and many changes were modest and between 1-3 percentage points in absolute value. Nineteen states experienced a 2016-to-2019 change of 3-5 percentage points in absolute value. These states include Alaska, Connecticut, Florida, Indiana, Iowa, Kansas, Louisiana, Missouri, Nebraska, Nevada, New Hampshire, New Mexico, New York, Ohio, Pennsylvania, South Carolina, South Dakota, Virginia, and Wyoming. Eight states experienced a 2016-to-2019 change of more than 5 percentage points in absolute value. These states include Arkansas, District of Columbia, Maryland, Mississippi, New Jersey, North Dakota, Texas, and West Virginia.

Table S3 shows the results of estimating our main models on specific preventive services that we considered as secondary outcomes. These outcomes include past-year dental cleaning, toothbrushing instructions, dental x-rays, fluoride treatment, and dental sealants. Our findings indicate a significant and positive association between the fee ratio and dental cleanings, toothbrushing instructions, and fluoride treatments. Associations with dental x-rays and sealants were not significant at conventional levels.

Table S4 replicates our main regression analysis for the preventive dental visit outcome including children of all ages rather than restricting to those ages 6-17. Overall, estimates were

© 2022 Lipton BJ et al. *JAMA Health Forum.*

similar to our main results when considering children of all ages. One important difference was that the estimate for any preventive dental visit for non-Hispanic Black children was statistically significant when including children of all ages (0.36 percentage point increase, P=0.04), which may be because of the increase in sample size.

Table S5 presents the results of our test for preexisting outcome trends. To conduct this analysis, we augmented our main regression model by including the future change in the fee ratio and its interaction with the Medicaid indicator as explanatory variables. For example, the future change in the fee ratio for 2016 observations was the difference between the 2017 and 2016 fee ratio in the state of residence. A significant coefficient on the interaction terms would suggest a violation of the parallel trends assumption required for difference-in-differences analysis. As shown in Table S5, none of these coefficients were statistically significant at conventional levels.

Table S6 presents the results of separate regressions that include observations from states that experienced a fee ratio change in 2017, 2018, and 2019, respectively, in addition to states that did not experience a change in any year. We defined a change in the fee ratio to be any change of at least 2 percentage points in absolute value. This value was selected to include most changes in the fee ratio while avoiding considering as changes very minor fluctuations that occur in most states in each year. It was necessary to define a threshold value for fee ratio changes to determine which states had changes in each year and which states could serve as control states.

We then re-estimated our main regression including all control variables except only including states with a change in the given year and states with no change in any year. The fee ratio was still included as a continuous variable. The first column of Table S6 shows the results of this exercise when only including states with a change of at least 2 percentage points in absolute value between 2016 and 2017, and states with no change larger than 2 percentage points

© 2022 Lipton BJ et al. *JAMA Health Forum.*

in any year as a comparison group. Estimates shown in the table are the coefficients on the interaction between the continuous fee ratio and the Medicaid indicator. Results were similar to our main analysis including all states both qualitatively and quantitatively. The estimates for the preventive dental visit variables remained statistically significant at conventional levels. The estimate for excellent oral health was similar in magnitude to our main results but was no longer statistically significant at conventional levels.

The second and third columns repeat this analysis for states with changes in 2018 and 2019, respectively. While estimates are no longer statistically significant, estimates for the dental visit variables remain positive and for the at least 2 visits variable, are of a similar magnitude. It is not surprising that these estimates are no longer statistically significant since it was necessary to exclude many states with policy changes, and also because it may take time for changes in the fee ratio to influence outcomes. For example, fee ratio changes that occur in 2019 may not have immediate effects on children’s dental visits and oral health in 2019, the final year of survey data in our study.

Overall, these results do not suggest that our main findings are biased by variation in treatment timing, Despite reducing our power by dividing states by the year of policy change and the need to define a specific threshold for policy changes, results comparing states with changes in 2017 to non-changer states are very similar to our main estimates and results for the 2018 and 2019 analyses are qualitatively similar for the dental visit variables.

Table S7 replicates our main analysis except limiting to states with a fee-for-service dental program according to a 2016 American Dental Association classification. These states include AK, AL, AR, CA, CT, DE, HI, IA, ID, IN, LA, MA, ME, MD, MT, NC, ND, NE, NH, OK, SC, SD, UT, VA, VT, WA, WI, and WY. We did not include DE and ID since these states

© 2022 Lipton BJ et al. *JAMA Health Forum.*

were missing Medicaid dental payment information. Results for the dental visit outcomes were statistically significant and larger in magnitude than our main analysis including all states. For example, when limiting to fee-for-service states, we estimated that a 1 percentage point increase in the fee ratio was associated with increases of 0.24 and 0.66 percentage points in at least 1 and at least 2 dental visits, respectively. By comparison, our main estimates for these outcomes were 0.18 and 0.27 percentage points, respectively. In contrast, the estimate for excellent oral health was somewhat smaller in magnitude and no longer statistically significant (P=0.16).

Table S8 replaces the continuous fee ratio with indicators for having a fee ratio above or below the 75th and 50th percentiles of the distribution, respectively. These indicators are significantly associated with increases in dental visits and excellent oral health, which is consistent with our main results. Also similar to our main results, associations with school absences are not significant at conventional levels.

Table S9 replicates our main analysis except the fee ratio is replaced with the inflation-adjusted Medicaid fee index in dollars. The consumer price index was used to adjust fees for inflation. Findings are qualitatively similar to our main results, as shown in the Table. We estimate that a $1 increase in the inflation-adjusted fee index is associated with increases of 0.31, 0.45, and 0.30 percentage points in at least 1 preventive visit, at least 2 preventive visits, and excellent oral health, respectively. Similar to our main analysis, associations with the school absences outcomes are negative but not statistically significant at conventional levels.

Table S10 presents the results of logistic regressions. Coefficients shown are odds ratios for the interaction between the continuous fee ratio and the Medicaid indicator. Estimates suggest positive and significant associations between the fee ratio and preventive dental visits (at least 1 and at least 2) and excellent oral health.

© 2022 Lipton BJ et al. *JAMA Health Forum.*

Table S11 presents the results of several sensitivity analyses. The first column shows the results of including a control for family income as a percent of the federal poverty level in our main models, the second shows the results of excluding 6 states that had a change in Medicaid adult dental coverage during our study period (CA, CO, CT, ID, IL, LA) based on data from the Center for Health Care Strategies, and the third shows the results of excluding foreign born children. In general, the results of these sensitivity tests were very similar to our main results.

Table S12 presents results when restricting the sample to Medicaid-enrolled children. These regressions were similar to our main analysis, except the coefficient of interest is the fee ratio rather than its interaction with Medicaid status. Estimates are larger in magnitude with larger standard errors relative to the analysis including a control group, which is not surprising given smaller sample sizes and lower power to detect significant effects. The coefficient estimates for the dental visit and excellent oral health outcomes are positive but no longer statistically significant. Estimates for the absences variables are negative, and the result for four or more school absences is statistically significant (P=0.008).

Finally, Figures S1-S4 plot trends in all outcomes except for at least 1 dental visit (main text Figure 2) in the 9 states that first had a change of at least 2 percentage points in the fee ratio in 2018 or 2019, as described in the main text. In general, trends appear similar for Medicaid-enrolled and privately-insured children, with the exception of at least 4 school absences. However, estimates have wide confidence intervals and it is not possible to rule out similar pretrends. These visual representations complement our regression-based analysis of pretrends shown in Appendix Table S5.

© 2022 Lipton BJ et al. *JAMA Health Forum.*

eTable 1. Association Between the Fee Ratio and Sample Characteristics, National Survey of Children's Health 2016-2019

Sample characteristic	Coefficient for fee ratio x Medicaid	P-value
Age in years	0.2485	0.64
Male	0.0372	0.61
Race and ethnicity		
Hispanic	-0.0068	0.96
Non-Hispanic White	-0.0369	0.76
Non-Hispanic Black	0.0176	0.89
Non-Hispanic Other Races	0.0261	0.74
US born	0.0343	0.59
Number of children in household		
1 child	0.0396	0.68
2 children	-0.1123	0.48
3 or more children	0.0727	0.64
Highest adult educational attainment		
High school diploma/GED or less	0.1006	0.24
Some college/Associates degree	0.0573	0.67
College or more education	-0.1579	0.20
Primary caregiver married or living with partner	-0.0184	0.91
Primary caregiver employed	-0.0698	0.46

Notes: Estimates shown in the table are in terms of percentage points and represent the results of a regressing each sample characteristic against the fee ratio and its interaction with the Medicaid coverage indicator, state, and year fixed effects. Estimates are the coefficient for the interaction term and represent the effect of a 1 percentage-point increase in the fee ratio. A separate regression was run for each sample characteristic as the left-hand-side variable. Models used sampling weights available from the National Survey of Children's Health and errors were clustered at the state level.

© 2022 Lipton BJ et al. *JAMA Health Forum.*

eTable 2. State-Level Fee Ratio Changes, 2016-2019

State	2016 fee ratio	2019 fee ratio	Percentage-point change in fee ratio
Alabama	0.4436904	0.4286633	-1.50271
Alaska	0.8138605	0.7699248	-4.39357
Arizona	0.5341711	0.5100223	-2.41488
Arkansas	0.5439956	0.4935295	-5.04661
California	0.3249517	0.3074094	-1.75423
Colorado	N/A	N/A	N/A
Connecticut	0.6311187	0.5832817	-4.7837
Delaware	N/A	N/A	N/A
District of Columbia	0.7319787	0.6635928	-6.83859
Florida	0.3469103	0.3144998	-3.24105
Georgia	0.4541866	0.4484071	-0.57795
Hawaii	0.3712658	0.3512233	-2.00425
Idaho	N/A	N/A	N/A
Illinois	0.5172662	0.508674	-0.85922
Indiana	0.5438124	0.4965391	-4.72733
Iowa	0.3972179	0.3619474	-3.52705
Kansas	0.4802005	0.4375617	-4.26388
Kentucky	0.7557048	0.7301102	-2.55946
Louisiana	0.5329608	0.4835185	-4.94423
Maine	0.3964864	0.3713023	-2.51841
Maryland	0.5884834	0.5335037	-5.49797
Massachusetts	0.4649461	0.4354136	-2.95325
Michigan	0.3112435	0.2841873	-2.70562
Minnesota	0.3013287	0.2745726	-2.67561
Mississippi	0.3020603	0.4230375	12.09772
Missouri	0.3965818	0.3614234	-3.51584
Montana	0.5201772	0.4915974	-2.85798
Nebraska	0.426489	0.3931204	-3.33686
Nevada	0.6661689	0.6210542	-4.51147
New Hampshire	0.5085679	0.4762646	-3.23033
New Jersey	0.0838692	0.1423675	5.84983
New Mexico	0.4830007	0.4502374	-3.27633
New York	0.5196757	0.4792918	-4.03839
North Carolina	0.4333093	0.4321191	-0.11902
North Dakota	0.6588653	0.6054412	-5.34241
Ohio	0.3545898	0.3237655	-3.08243
Oklahoma	0.4539682	0.4295464	-2.44218
Oregon	0.3406985	0.3545632	1.38647
Pennsylvania	0.4166392	0.3842622	-3.2377
Rhode Island	0.2814714	0.2635928	-1.78786

© 2022 Lipton BJ et al. *JAMA Health Forum.*

South Carolina	0.5025948	0.4556393	-4.69555
South Dakota	0.6116523	0.5735633	-3.8089
Tennessee	N/A	N/A	N/A
Texas	0.5753447	0.5219705	-5.33742
Utah	0.4272293	0.4401642	1.29349
Vermont	0.462934	0.4335293	-2.94047
Virginia	0.4901667	0.4443723	-4.57944
Washington	0.3140282	0.2970756	-1.69526
West Virginia	0.5698159	0.5165802	-5.32357
Wisconsin	0.3270376	0.2998111	-2.72265
Wyoming	0.5875183	0.5477676	-3.97507

Notes: The table shows the ratio of Medicaid payment rates to dentist charges for an index of services as described in Appendix A. Medicaid payment rates were obtained from state fee schedules in each year. Dentist charges for the same services were obtained from the American Dental Association. Medicaid payment rates were not available in CO, DE, ID, and TN. The "Percentage-point change in the fee ratio" is the difference between the 2019 and 2016 fee ratio in each state in terms of percentage points.

© 2022 Lipton BJ et al. *JAMA Health Forum.*

eTable 3. Difference-in-Differences Estimates of the Association Between the Fee Ratio and Specific Preventive Services, National Survey of Children's Health 2016-2019

Outcome	Difference-in-differences estimate (95% CI)	P value
Dental cleaning	0.2149 (0.0991 to 0.3307	0.001
Toothbrushing instructions	0.3354 (0.2382 to 0.4325)	<0.001
Dental x-rays	0.0798 (-0.0412 to 0.2008)	0.19
Fluroride treatment	0.1546 (0.0319 to 0.2773)	0.015
Dental sealants	0.0590 (-0.0517 to 0.1697)	0.29

Notes: Estimates shown in the table are in terms of percentage points. Difference-in-differences estimates represent the effect of a 1 percentage-point increase in the fee ratio. Models include state and year fixed effects in addition to child and time-varying state-level controls (unemployment rate, Medicaid managed care penetration rate, dentist supply per capita, Medicaid income eligibility limit for working parents). Estimates are weighted and model errors are clustered at the state level.

eTable 4. Difference-in-Differences Estimates of the Association Between the Fee Ratio and Children's Preventive Dental Visits for Children of All Ages, National Survey of Children's Health 2016-2019

Outcome	Weighted percent	Difference-in-differences estimate (95% CI)	P value
**Any preventive dental visit**			
All	75	0.1981 (0.0774 to 0.3187)	0.002
Subgroup analysis by race and ethnicity			
Non-Hispanic White [Ref]	74	0.1271 (-0.0038 to 0.2580)	0.06
Hispanic	76	0.2115 (0.0514 to 0.3717)	0.011
Non-Hispanic Black	75	0.3627 (0.0186 to 0.7068)	0.04
Non-Hispanic Other Race	73	-0.0954 (-0.2741 to 0.0833)	0.29
Subgroup analysis by sex			
Male [Ref]	75	0.2536 (0.0684 to 0.4389)	0.008
Female	74	0.1328 (0.0249 to 0.2408)	0.017
**Two or more preventive dental visits**			
All	40	0.2239 (0.0564 to 0.3914)	0.01
Subgroup analysis by race and ethnicity			
Non-Hispanic White [Ref]	41	0.0782 (-0.1229 to 0.2793)	0.44
Hispanic	41	0.5013 (0.2603 to 0.7423)	<0.001
Non-Hispanic Black	36	0.0581 (-0.2960 to 0.4122)	0.74
Non-Hispanic Other Race	37	-0.2071 (-0.0655 to 0.4797)	0.13
Subgroup analysis by sex			
Male [Ref]	39	0.2142 (0.0114 to 0.4170)	0.04
Female	40	0.2371 (-0.0341 to 0.5083)	0.09

Notes: Estimates shown in the table are in terms of percentage points. Weighted rates are for Medicaid-enrolled children. Difference-in-differences estimates represent the effect of a 1 percentage-point increase in the fee ratio. Models include state and year fixed effects in addition to child and time-varying state-level controls (unemployment rate, Medicaid managed care penetration rate, dentist supply per capita, Medicaid income eligibility limit for working parents). Estimates are weighted and model errors are clustered at the state level.

© 2022 Lipton BJ et al. *JAMA Health Forum.*

eTable 5. Test for Preexisting Differences in Outcome Trends, National Survey of Children's Health 2016-2019

Outcome	Coefficient estimate for trend	P-value
Any preventive dental visit	-0.4811	0.33
Two or more preventive dental visits	-0.8091	0.25
Excellent condition of teeth	-0.3067	0.62
Four or more school absences	0.5974	0.39
Seven or more school absences	0.2942	0.59

Notes: Estimates shown in the table are the coefficient for the interaction between the future change in the fee ratio and Medicaid status, as described in Appendix D and in the main text.

© 2022 Lipton BJ et al. *JAMA Health Forum.*

eTable 6. Difference-in-Differences Estimates of the Association Between the Fee Ratio and Preventive Dental Visits, Oral Health, and School Absences by Year of Policy Change, National Survey of Children's Health 2016-2019

	Year of fee change		
**Outcome**	**2017**	**2018**	**2019**
Any preventive dental visit	**0.1678 (0.0436 to 0.2920)**	0.1074 (-0.0421 to 0.2569)	0.0859 (-0.0293 to 0.2011)
Two or more preventive dental visits	**0.2958 (0.0345 to 0.5572)**	0.2205 (-0.1100 to 0.5510)	0.2266 (-0.0845 to 0.5376)
Excellent condition of teeth	0.1857 (-0.0151 to 0.3866)	-0.0041 (-0.1891 to 0.1810)	0.0275 (-0.1758 to 0.2307)
Four or more school absences	-0.0436 (-0.1841 to 0.0969)	-0.0083 (-0.1928 to 0.1761)	-0.0118 (-0.1928 to 0.1692)
Seven or more school absences	-0.0787 (-0.1842 to 0.0268)	-0.0729 (-0.2217 to 0.0758)	-0.0702 (-0.2177 to 0.0772)

Notes: Estimates shown in the table are in terms of percentage points. The estimates from each column come from separate regression models where the sample is restricted to states with a fee ratio change in the year indicated and states with no fee ratio change in any year, as described in Appendix D. Difference-in-differences estimates represent the effect of a 1 percentage-point increase in the fee ratio. Models include state and year fixed effects in addition to child and time-varying state-level controls (unemployment rate, Medicaid managed care penetration rate, dentist supply per capita, Medicaid income eligibility limit for working parents). Estimates are weighted and model errors are clustered at the state level.

© 2022 Lipton BJ et al. *JAMA Health Forum.*

eTable 7. Difference-in-Differences Estimates of the Association Between the Fee Ratio and Preventive Dental Visits, Oral Health, and School Absences in States With a Fee-for-Service Dental Program, National Survey of Children's Health 2016-2019

Outcome	Difference-in-differences estimate (95% CI)	P value
Any preventive dental visit	0.2396 (0.0849 to 0.3942)	0.004
Two or more preventive dental visits	0.6647 (0.3875 to 0.9420)	<0.001
Excellent condition of teeth	0.1136 (-0.0458 to 0.2731)	0.16
Four or more school absences	-0.2119 (-0.3577 to -0.0661)	0.006
Seven or more school absences	-0.1730 (-0.2950 to -0.0509)	0.007

Notes: Estimates shown in the table are in terms of percentage points. Difference-in-differences estimates represent the effect of a 1 percentage-point increase in the fee ratio. Models include state and year fixed effects in addition to child and time-varying state-level controls (unemployment rate, Medicaid managed care penetration rate, dentist supply per capita, Medicaid income eligibility limit for working parents). Estimates are weighted and model errors are clustered at the state level. The sample was restricted to residents of states with a fee-for-service dental program based on the American Dental Association's 2016 classification, as described in Appendix D (see Gupta N, Yarbrough C, Vujicic M, Blatz A, Harrison B. Medicaid Fee-For-Service Reimbursement Rates for Child and Adult Dental Care Services for all States, 2016. American Dental Association, Health Policy Institute, 2017.).

© 2022 Lipton BJ et al. *JAMA Health Forum.*

eTable 8. Difference-in-Differences Estimates of the Association Between the Fee Ratio Percentile and Preventive Dental Visits, Oral Health, and School Absences, National Survey of Children's Health 2016-2019

Outcome	75th percentile indicator, DiD	P value	50th percentile indicator, DiD	P value
Any preventive dental visit	6.23	<0.001	3.27	0.02
Two or more preventive dental visits	5.70	0.003	8.45	<0.001
Excellent condition of teeth	5.99	0.01	4.70	0.004
Four or more school absences	-1.99	0.30	-0.74	0.57
Seven or more school absences	-2.17	0.11	-1.62	0.15

Notes: Estimates shown in the table are in terms of percentage points and represent the effects of changing the fee ratio from being below to being above the indicated percentile (75th or 50th). Models include state and year fixed effects in addition to child and time-varying state-level controls (unemployment rate, Medicaid managed care penetration rate, dentist supply per capita, Medicaid income eligibility limit for working parents). Estimates are weighted and model errors are clustered at the state level.

© 2022 Lipton BJ et al. *JAMA Health Forum.*

eTable 9. Difference-in-Differences Estimates of the Association Between the Inflation-Adjusted Medicaid Fee Index and Preventive Dental Visits, Oral Health, and School Absences, National Survey of Children's Health 2016-2019

Outcome	Difference-in-differences estimate (95% CI)	P value
Any preventive dental visit	0.3077 (0.1377 to 0.4776)	0.001
Two or more preventive dental visits	0.4534 (0.0648 to 0.8419)	0.02
Excellent condition of teeth	0.2962 (0.0236 to 0.5687)	0.03
Four or more school absences	-0.1196 (-0.3243 to 0.0852)	0.25
Seven or more school absences	-0.1516 (-0.3070 to 0.0038)	0.06

Notes: Estimates shown in the table are in terms of percentage points given a $1 increase in the inflation-adjusted Medicaid fee index. Models include state and year fixed effects in addition to child and time-varying state-level controls (unemployment rate, Medicaid managed care penetration rate, dentist supply per capita, Medicaid income eligibility limit for working parents). Estimates are weighted and model errors are clustered at the state level.

© 2022 Lipton BJ et al. *JAMA Health Forum.*

eTable 10. Difference-in-Differences Estimates of the Association Between the Fee Ratio and Preventive Dental Visits, Oral Health, and School Absences, Logit Regression Models, National Survey of Children's Health 2016- 2019

Outcome	Difference-in-differences estimate - odds ratio (95% CI)	P value
Any preventive dental visit	1.0140 (1.0051 to 1.0230)	0.002
Two or more preventive dental visits	1.0113 (1.0017 to 1.0210)	0.02
Excellent condition of teeth	1.0091 (1.0014 to 1.0169)	0.02
Four or more school absences	0.9963 (0.9896 to 1.0031)	0.29
Seven or more school absences	0.9913 (0.9815 to 1.0012)	0.09

Notes: Estimates shown in the table are odds ratios from logistic regression models. Difference-in-differences estimates represent the effect of a 1 percentage-point increase in the fee ratio. Models include state and year fixed effects in addition to child and time-varying state-level controls (unemployment rate, Medicaid managed care penetration rate, dentist supply per capita, Medicaid income eligibility limit for working parents). Estimates are weighted and model errors are clustered at the state level.

© 2022 Lipton BJ et al. *JAMA Health Forum.*

eTable 11. Difference-in-Differences Estimates of the Association Between the Fee Ratio and Preventive Dental Visits, Oral Health, and School Absences, Various Controls and Sample Exclusions, National Survey of Children's Health 2016-2019

Outcome	Difference-in-differences estimate (95% CI)		
Any preventive dental visit	0.1874 (0.0758 to 0.2990)	0.2173 (0.1045 to 0.3302)	0.1818 (0.0727 to 0.2910)
Two or more preventive dental visits	0.2794 (0.0398 to 0.5191)	0.2398 (0.0468 to 0.4327)	0.2804 (0.0257 to 0.5350)
Excellent condition of teeth	0.1875 (0.0086 to 0.3662)	0.1596 (-0.0292 to 0.3484)	0.1759 (-0.0090 to 0.3607)
Four or more school absences	-0.0724 (-0.2053 to 0.0606)	-0.0293 (-0.1627 to 0.1042)	-0.0978 (-0.2369 to 0.0413)
Seven or more school absences	-0.0958 (-0.1923 to 0.0006)	-0.0612 (-0.1538 to 0.0313)	-0.0902 (-0.1962 to 0.0157)
**Model/Exclusion**	Add income control	Exclude adult dental states	Exclude foreign born

Notes: Estimates shown in the table are in terms of percentage points. Difference-in-differences estimates represent the effect of a 1 percentage-point increase in the fee ratio. Each column represents a separate set of regressions that incorporate the control or exclusion as indicated in the table. Models include state and year fixed effects in addition to child and time-varying state-level controls (unemployment rate, Medicaid managed care penetration rate, dentist supply per capita, Medicaid income eligibility limit for working parents). Estimates are weighted and model errors are clustered at the state level.

© 2022 Lipton BJ et al. *JAMA Health Forum.*

eTable 12. Difference-in-Differences Estimates of the Association Between the Fee Ratio and Preventive Dental Visits, Oral Health, and School Absences Excluding the Privately Insured Control Group, National Survey of Children's Health 2016-2019

Outcome	Difference-in-differences estimate - percentage points (95% CI)	P value
Any preventive dental visit	0.3448 (-0.3357 to 1.0253)	0.31
Two or more preventive dental visits	0.7297 (-0.2068 to 1.6482)	0.13
Excellent condition of teeth	0.3862 (-0.4971 to 1.2696)	0.38
Four or more school absences	-0.8091 (-1.3950 to -0.2232)	0.008
Seven or more school absences	-0.2903 (-0.8550 to 0.2745)	0.31

Notes: Estimates shown in the table are in terms of percentage points and are for the coefficient on the continuous fee ratio variable. Difference-in-differences estimates represent the effect of a 1 percentage-point increase in the fee ratio. Models include state and year fixed effects in addition to child and time-varying state-level controls (unemployment rate, Medicaid managed care penetration rate, dentist supply per capita, Medicaid income eligibility limit for working parents). Estimates are weighted and model errors are clustered at the state level. The sample was restricted to publicly insured children.

© 2022 Lipton BJ et al. *JAMA Health Forum.*

eFigure 1. Trends in at Least 2 Dental Visits in States With a 2018-2019 Fee Ratio Change

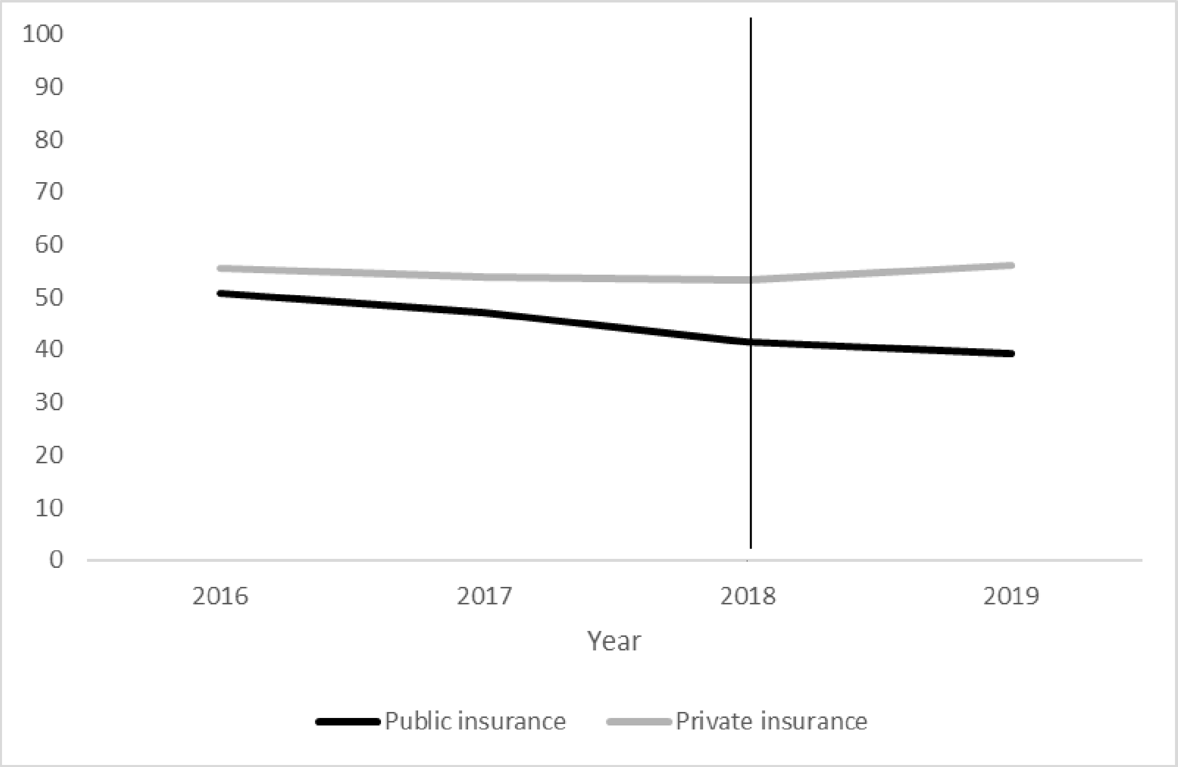

Source: Authors' analysis of the 2016-2019 NSCH. Notes: Outcome rates are weighted using sampling weights available from NSCH. The sample includes children in the main study sample that resided in 1 of 9 states that first had a year-over-year fee ratio change of at least 2 percentage points in 2018 or 2019. These states include Alaska, Arizona, Connecticut, Montana, Mississippi, North Carolina, Oregon, South Dakota, and Utah. The sample includes 3420 Medicaid-enrolled children and 2923 privately insured children.

© 2022 Lipton BJ et al. *JAMA Health Forum.*

eFigure 2. Trends in Excellent Oral Health in States With a 2018-2019 Fee Ratio Change

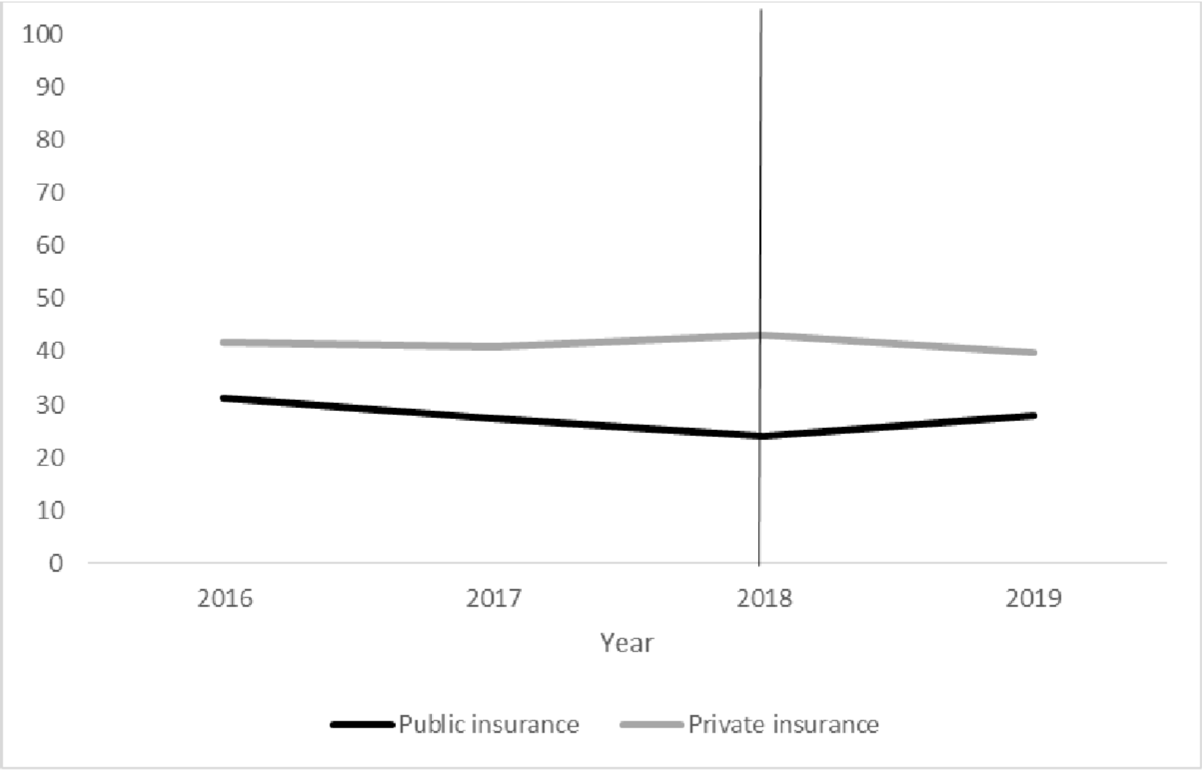

Source: Authors' analysis of the 2016-2019 NSCH. Notes: Outcome rates are weighted using sampling weights available from NSCH. The sample includes children in the main study sample that resided in 1 of 9 states that first had a year-over-year fee ratio change of at least 2 percentage points in 2018 or 2019. These states include Alaska, Arizona, Connecticut, Montana, Mississippi, North Carolina, Oregon, South Dakota, and Utah. The sample includes 3420 Medicaid-enrolled children and 2923 privately insured children.

© 2022 Lipton BJ et al. *JAMA Health Forum.*

eFigure 3. Trends in at Least 4 School Absences in States With a 2018-2019 Fee Ratio Change

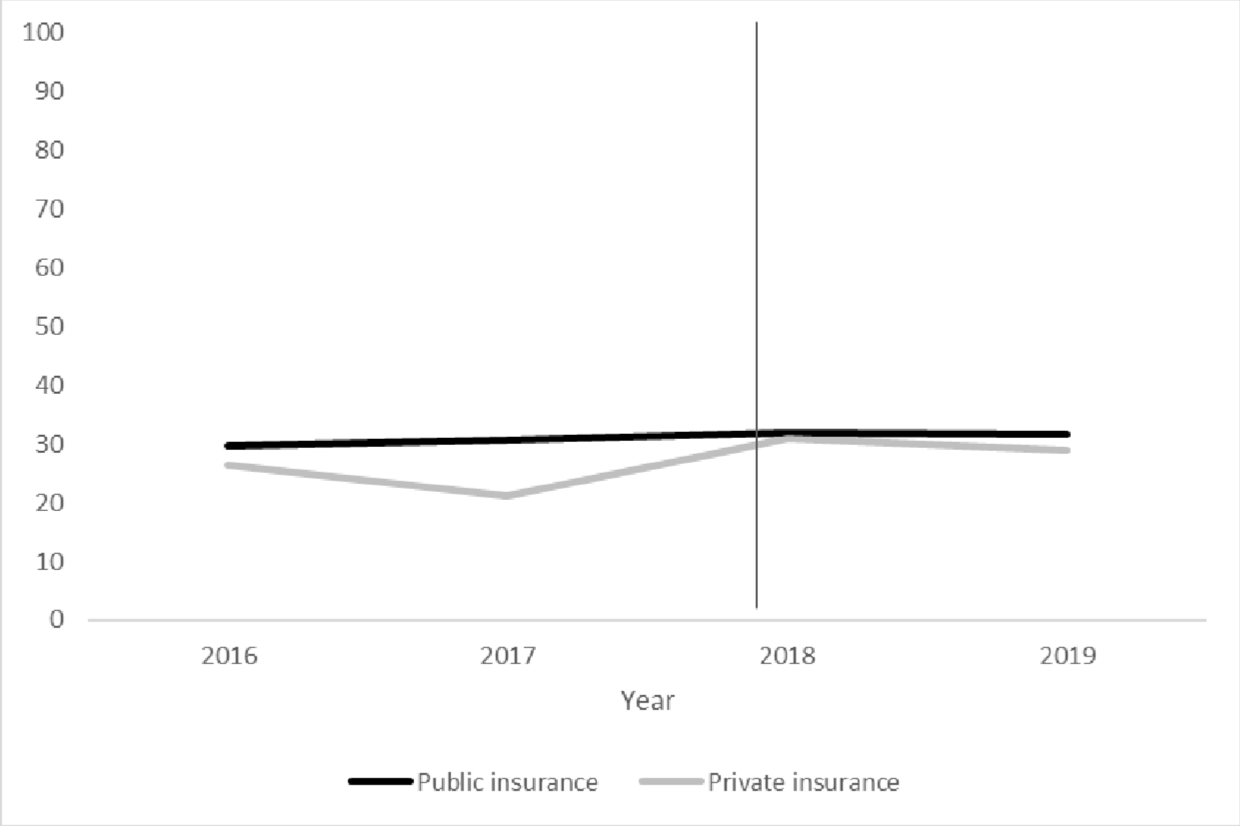

Source: Authors' analysis of the 2016-2019 NSCH. Notes: Outcome rates are weighted using sampling weights available from NSCH. The sample includes children in the main study sample that resided in 1 of 9 states that first had a year-over-year fee ratio change of at least 2 percentage points in 2018 or 2019. These states include Alaska, Arizona, Connecticut, Montana, Mississippi, North Carolina, Oregon, South Dakota, and Utah. The sample includes 3420 Medicaid-enrolled children and 2923 privately insured children.

eFigure 4. Trends in at Least 7 School Absences in States With a 2018-2019 Fee Ratio Change

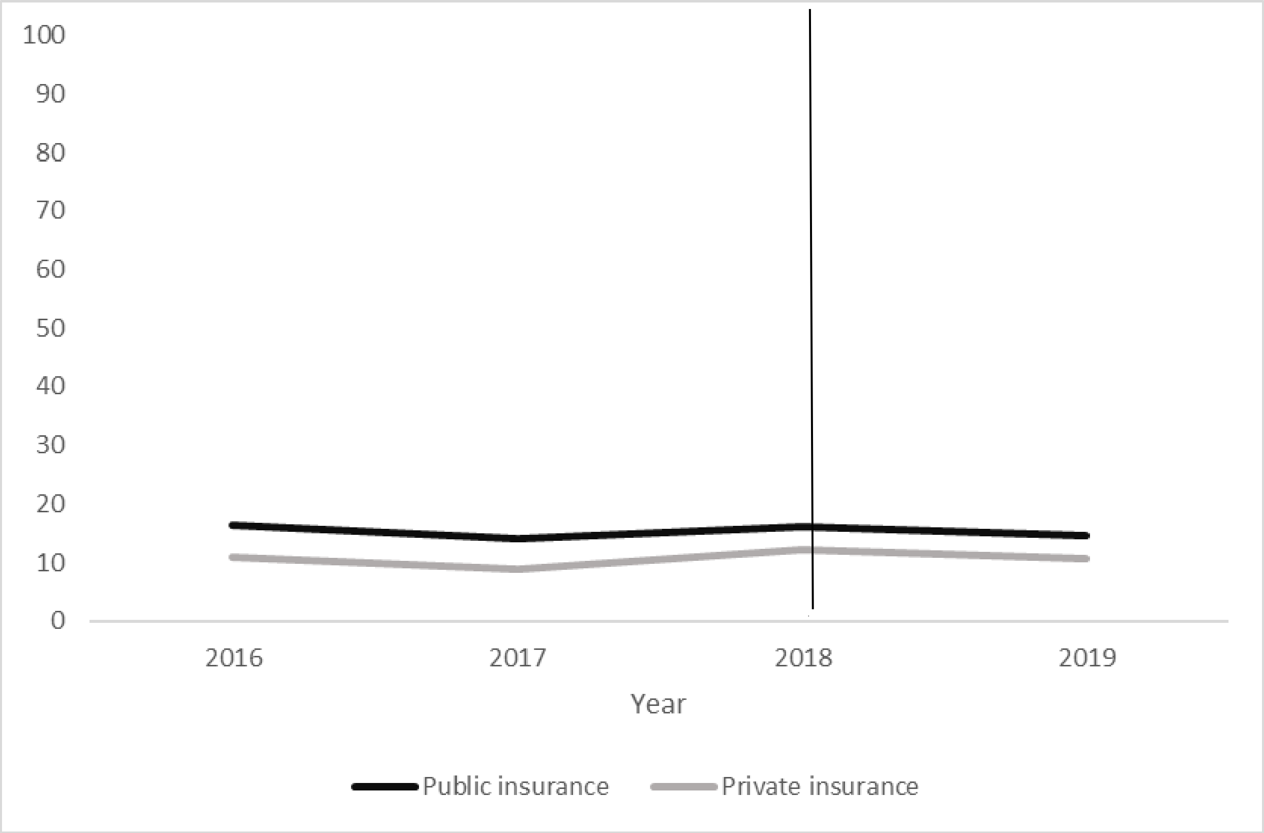

Source: Authors' analysis of the 2016-2019 NSCH. Notes: Outcome rates are weighted using sampling weights available from NSCH. The sample includes children in the main study sample that resided in 1 of 9 states that first had a year-over-year fee ratio change of at least 2 percentage points in 2018 or 2019. These states include Alaska, Arizona, Connecticut, Montana, Mississippi, North Carolina, Oregon, South Dakota, and Utah. The sample includes 3420 Medicaid-enrolled children and 2923 privately insured children.

© 2022 Lipton BJ et al. *JAMA Health Forum.*
